# *Cryptosporidium* infections in sheep farms from Italy

**DOI:** 10.1007/s00436-020-06947-2

**Published:** 2020-11-03

**Authors:** G. Dessì, C. Tamponi, A. Varcasia, G. Sanna, A. P. Pipia, S. Carta, F. Salis, P. Díaz, A. Scala

**Affiliations:** 1 Laboratory of Parasitology, Veterinary Teaching Hospital, Department of Veterinary Medicine, University of Sassari, Parassitologia Veterinaria, Via Vienna 2, 07100 Sassari, Italy; 2 Investigation in Animal Health: Galicia (INVESAGA Group), School of Veterinary Medicine, Universidad de Santiago de Compostela, Lugo, Spain

**Keywords:** *Cryptosporidium*, Lambs, Sheep, Zoonosis, Italy

## Abstract

Cryptosporidiosis is recognized as being a significant cause of gastrointestinal illness due to its wide range of vertebrate hosts, including humans. Infection with *Cryptosporidium* spp. is especially common in young domestic ruminants (calves, lambs and goat kids) and has been associated with economic losses worldwide. In contrast to cattle, to date, detailed studies on *Cryptosporidium* infections in sheep from Europe are still limited; thus, their importance as reservoirs of *Cryptosporidium* species with implications on animal and public health still needs to be clarified. This study evaluates the prevalence and zoonotic potential of *Cryptosporidium* spp. in sheep farms in Italy. A total of 915 individual faecal samples divided into three different animal categories were collected from 61 sheep farms. Each sample was examined by microscopy of faecal smears stained by modified Ziehl-Neelsen and by biomolecular techniques. *Cryptosporidium* oocysts were detected in 10.1% of the animals examined and in 34.4% of the farms. The prevalence of *Cryptosporidium* spp. was significantly higher (χ^2^ = 51.854; *P* < 0.001) in diarrhoeic samples than in pasty or normal faeces. Genotype analyses showed the presence of two *Cryptosporidium* species: *C. parvum* and *C. ubiquitum*. Subtyping analysis of *C. parvum* isolates revealed the presence of subtypes IIa15G2R1 and IIdA20G1 and of subtype XIIa for *C. ubiquitum*. These findings have public health implications since both *Cryptosporidium* species identified are considered zoonotic, and *C. parvum* is the second-most common *Cryptosporidium* species infecting humans. Our data reveal that lambs, especially those excreting diarrhoeic faeces, may be important reservoirs of *Cryptosporidium.* We also highlight the need to establish adequate control and monitoring programmes for the control of this infection in sheep farms primarily through coprological monitoring.

## Introduction

Cryptosporidiosis is a global disease caused by apicomplexan parasites belonging to the genus *Cryptosporidium*, which are recognized as a significant source of gastrointestinal illness for a wide range of vertebrate hosts, including humans (Xiao, 2010, Ryan et al., 2016). Molecular investigations have identified nearly 40 species and over 50 genotypes, of which *Cryptosporidium hominis* and *Cryptosporidium parvum* are the most prevalent in humans, causing asymptomatic or mild-to-severe gastrointestinal diseases (Ryan et al., 2014, Feng et al., 2018, Firoozi et al., 2019, Roellig and Xiao, 2020).

Among livestock, ruminants are considered to be important reservoirs of both host-specific and zoonotic *Cryptosporidium* species as they shed a large number of oocysts that cause environmental contamination (Xiao, 2010, Santin, 2020). In particular, cattle have been considered a health risk to humans due to the potential source of cryptosporidiosis (Santin, 2020).

However, little is known about the disease and there are contrasting opinions about the transmission route and the role played by different animal species, such as sheep and goats, in the epidemiology of human infections (Ryan et al., 2005, Broglia et al. 2008, Xiao, 2010, Cacciò et al., 2013). Nevertheless, sheep and/or goats appear to harbour zoonotic species or genotypes of the protozoan, suggesting that sheep and/or goats should be considered as epidemiologically significant reservoirs (Geurden et al., 2008, Mueller-Doblies et al., 2008,Quìlez et al., 2008).

*Cryptosporidium* infections, due primarily to zoonotic *C. parvum*, are especially common in young domestic ruminants (calves, lambs and goat kids) and are associated with economic losses due to the cost of veterinary care and the clinical symptoms including diarrhoea, dehydration, delayed growth and weight loss, often leading to death (Castro-Hermida et al., 2002, Ye et al., 2013, Mammeri et al., 2019, Hatam-Nahavandi et al., 2019, Santin, 2020).

Sheep can be infected with a number of *Cryptosporidium* species. Of these, *C. parvum*, *C. xiaoi* and *C. ubiquitum* are the most common, while *C. hominis*, *C. andersoni*, *C. bovis*, *C. scrofarum, C. suis, C. fayeri* and *C. canis* have been sporadically reported (Pritchard et al., 2008, Paraud and Chartier, 2012, Koinari et al., 2014, Squire et al., 2017, Díaz et al., 2010, 2015, 2018a, Zhang et al., 2018).

The presence of *Cryptosporidium* spp. in sheep worldwide shows variations in the prevalence related to factors such as the age and health of the sheep. In healthy animals, for example, lower prevalence rates (0.9 to 14.3%) have been reported in adult sheep (Li et al., 2016, Díaz et al., 2018a,; Holsback et al., 2018, Qi et al., 2019) than in lambs (6.0% to 20.3%) (Díaz et al., 2018a; Majeed et al., 2018; Holsback et al., 2018). However, the highest percentages of infection have been recorded in lambs with diarrhoea, showing prevalences ranging from 24.16 to 100% (Papanikolopoulou et al., 2018, Mammeri et al., 2019, Rabee et al., 2020).

In Italy, *Cryptosporidium* spp. have been reported in humans (Cacciò et al., 2013) and in domestic animals such as dogs (Cervone et al., 2019), cats (Sauda et al., 2019), horses (Lanci et al., 2018) and cattle (Díaz et al., 2018b). However, data on *Cryptosporidium* infections in sheep in Italy are limited to a single survey carried out in Abruzzo, Central Italy, which reported that 17.45% of lambs were seropositive to *Cryptosporidium* (Paoletti et al., 2009). Despite the lack of data on the spread of *Cryptosporidium* infection in sheep in Italy, lambs have been identified as a source of zoonotic transmission to humans (Cacciò et al., 2013).

Unlike for cattle, detailed studies on *Cryptosporidium* infections in sheep in Europe are still limited, and the majority of data concern the prevalence in diarrhoeic lambs (Quílez et al., 2008, Pritchard et al., 2008, Paoletti et al., 2009, Cacciò et al., 2013, Díaz et al., 2010, 2015). However, there are few data on the occurrence of this protozoan in healthy sheep, especially post-weaned and adults; thus, their importance as reservoirs of *Cryptosporidium* species with implications for animal and public health still needs to be clarified.

Sardinia hosts 45% of the entire sheep stock in Italy and almost 4% of the European sheep population (Varcasia et al., 2020). One recent study reported a prevalence of 38.8% for *Cryptosporidium* spp. in calves in Sardinia (Díaz et al., 2018b). The present study contributes to global knowledge on *Cryptosporidium* infections in sheep, by evaluating the prevalence and zoonotic potential of *Cryptosporidium* spp. in sheep farms in Italy.

## Material and methods

### Sample collection and microscopic analysis

From November 2015 to February 2016, a total of 61 sheep farms located in the four provinces of Sardinia (Cagliari, Sassari, Oristano, Nuoro) were investigated, and a total of 915 individual faecal samples were collected. All the farms selected adopt a semi-intensive management system (pasture or range grazing, supplementary feeding mainly on commercial and/or cereal fodder) and no artificial suckling of lambs; both sheep and lambs share the same night shelter during the lambing season. A total of 15 samples from each flock were collected.

Five samples from each animal group were collected as follows: lambs aged 5–30 days (group 1), sheep from parturition to 30 days (group 2) and sheep within the 30 days before parturition (group 3). After collection from the rectum, each faecal sample was macroscopically examined to establish the faecal consistency, which was classified as normal (well-formed faeces), pasty (soft, not well formed) or diarrhoeic (liquid faeces). Faecal samples were also checked for the presence of mucus or blood.

Samples were then transported in a cool box to the Parasitology Laboratory at the Veterinary Teaching Hospital of the University of Sassari, Italy, and then stored at 4 °C until examination within 24 h of collection. Faecal samples were then examined for the presence of *Cryptosporidium* spp. oocysts by microscopy of faecal smears stained using the modified Ziehl-Neelsen technique according to Angus (1987). The intensity of oocyst shedding was evaluated semi-quantitatively according to the average number of oocysts in 10 fields at × 400 magnification, expressed as oocysts per field (OPF) as described by Castro-Hermida et al. (2002). The mean intensity (MI) was calculated considering the OPF arithmetic average of the total number of the infected animals, considering the groups and faecal consistency. All microscopic positive samples were stored at − 20 °C for further molecular characterization.

### Genotype analysis

Following three cycles of freezing with liquid nitrogen at − 196 °C (1 min) and thawing at 100 °C (5 min), total DNA was extracted from 200 mg of faeces using the QIAamp DNA Stool Mini Kit (QIAGEN, Hilden, Germany) in accordance with the manufacturer’s instructions.

*Cryptosporidium* species were determined by nested PCR of a ≈ 830 bp fragment of the SSU rRNA gene and restriction fragment length polymorphism (RFLP) analysis of the secondary PCR products with the endonucleases *Ssp*I, *Vsp*I and *Mbo*II, using the same primers and protocol previously described (Xiao et al., 1999, Jiang et al., 2005). Two samples of each *Cryptosporidium* species identified were sequenced in order to confirm the RFLP results.

Species were assigned by comparing RFLP profiles to those reported by Xiao and Ryan (2008). Subtyping analysis of samples identified as *C. parvum* and *C. ubiquitum* at the SSU rRNA locus was performed using a nested PCR followed by sequence analysis of the 60 kDa glycoprotein (gp60) fragment (800–850 bp and 950 bp, respectively), as previously described (Alves et al., 2003, Li et al., 2014).

PCR products were purified using Nucleospin Gel and PCR Clean Up (Macherey-Nagel GmbH & Co. KG, Düren, Germany) and sent to an external sequencing service (Eurofins Genomics, Ebersberg, Germany). The sequences obtained were compared with those on the NCBI database using BLAST (http://www.ncbi.nlm.nih.gov/BLAST/). *Cryptosporidium parvum* subtypes were named according to the nomenclature described by Sulaiman et al. (2005).

### Statistical analysis

All data were recorded on a spreadsheet (Microsoft Excel®^,^ Microsoft Corp., Redmond, WA) and subsequently processed using MINITAB v. 12.1 (Minitab Inc., State College, PA, USA) and Epi-info 6.04 (CDC, Atlanta, GA, USA). A chi-square test (χ^2^) was used to determine the statistical differences in prevalence rates of *Cryptosporidium* spp. infection between animal groups and those between animal groups considering faecal consistency. Results were considered statistically significant when *P* < 0.05. Odds ratios (OR) were calculated to evaluate the strength of association for *Cryptosporidium* spp. infection. The existence of significant differences in the average number of oocysts considering faecal consistency and the groups was analysed using the non-parametric Kruskal-Wallis test.

## Results

### Microscopic analysis

*Cryptosporidium* oocysts were detected in 10.1% (CI95%: 8.1–12.1) of the animals examined and in 34.4% (CI95%: 22.5–46.3) of the farms, in most cases involving only the lamb group (61.9% of positive farms). Only one positive farm (4.8%; 1/21) showed *Cryptosporidium* oocysts in all animal groups.

The percentage of animals shedding *Cryptosporidium* oocysts and the MI considering both animal groups and faecal consistency are summarized in Table 1. No samples presented mucus or blood. The prevalence found in pre-weaned lambs was significantly higher than those found in group 2 (χ^2^ = 14.52; *P* < 0.001) and group 3 (χ^2^ = 12.35; P < 0.001). No significant differences were found between ewes belonging to groups 2 and 3 (χ^2^ = 0.10; *P* = 0.719).

**Table 1:** Prevalence and mean intensity (MI) of *Cryptosporidium* spp. in sheep and lambs from Sardinia (Italy) in terms of animal group and faecal consistency

	Group 1		Group 2		Group 3		Total
	Positives/total (%)	MI (stand. dev.)	Positives/total (%)	MI (stand. dev.)	Positives/total (%)	MI (stand. dev.)	Positive/total (%)
Normal	10/140 (7.1)	13.00 (29.1)	7/154 (4.5)	22.29 (33.5)	10/181 (5.5)	5.80 (10.9)	27/475 (5.7)
Pasty	14/85 (16.4)	9.29 (16.4)	13/150 (8.6)	9.26 (15.1)	12/122 (9.8)	5.08 (4.9)	39/357 (10.9)
Diarrhoeic	26/80 (32.5)	25.23 (51.0)	0/1 (0)	0	0/2 (0)	0	26/83 (31.3)
Total	50/305 (16.4)	18.24 (40.53)	20/305 (6.6)	14.04 (23.18)	22/305 (7.2)	5.51 (7.97)	92/915 (10.1)

The prevalence of *Cryptosporidium* spp. decreased with increased faecal consistency and was significantly higher (χ^2^ = 51.854; *P* < 0.001) in diarrhoeic samples than in pasty or normal faeces (Table 1). Most individuals from all animal groups excreted normal or pasty faeces; in contrast, 26.2% (95% CI: 21.3–31.1) of samples from lambs were diarrhoeic, whereas only 0.5% (95% CI: − 0.1 to 1.1) of ewes were diarrhoeic.

When the possible influence of faecal consistency on the prevalence was analysed in terms of the different animal groups, only significant differences were found in group 1 (χ^2^ = 23.88; degrees of freedom (df) = 2, *P* < 0.001) where the probability of finding *Cryptosporidium* oocysts (OR) in pasty and diarrhoeic faeces was 2.56- and 6.26-fold higher, respectively, than in normal faeces.

The MI was higher in group 1 (18.24 ± 40.53 standard deviation—SD) than in group 2 (14.04 ± 23.18 SD) and group 3 (5.51 ± 7.97 SD), but no statistically significant differences (*P* > 0.05) were recorded using the Kruskal-Wallis test (Table 1). In addition, the MI detected in diarrhoeic faeces (25.23 ± 51.01 SD) was higher than in normal (12.74 ± 25.22 SD) and pasty (8.10 ± 13.25 SD) samples (Table 1); however, these differences were not significant using the Kruskal-Wallis test (*P* > 0.05).

### Genotype analysis

Of the 92 *Cryptosporidium* spp.–positive faecal samples at microscopy, only 15 were successfully genotyped at SSU rRNA because of the small amount of faecal material available and the low parasite loading. These *Cryptosporidium* isolates were identified as *C. parvum* (11) and *C. ubiquitum* (4). Subtyping analysis of *C. parvum* isolates revealed the presence of subtypes IIa15G2R1 and IIdA20G1 and of subtype XIIa for *C. ubiquitum*. Unfortunately, the subtyping of five *C. parvum* isolates was not successful. Detailed data are summarized in Table 2.

**Table 2:** Species and subtypes of *Cryptosporidium* spp. identified in the lamb samples examined

Id sample	Animal group	Faecal consistency	Species	Subtypes
1	1	Diarrhoeic	*C. ubiquitum*	XIIa
2	1	Pasty	*C. ubiquitum*	XIIa
3	1	Diarrhoeic	*C. parvum*	FAILED
4	1	Diarrhoeic	*C. parvum*	FAILED
5	1	Normal	*C. parvum*	IIaA15G2R1
6	1	Diarrhoeic	*C. parvum*	IIaA15G2R1
7	1	Diarrhoeic	*C. parvum*	FAILED
8	1	Diarrhoeic	*C. parvum*	IIaA15G2R1
9	1	Diarrhoeic	*C. parvum*	IIdA20G1
10	1	Diarrhoeic	*C. parvum*	FAILED
11	1	Diarrhoeic	*C. parvum*	FAILED
12	1	Diarrhoeic	*C. parvum*	IIaA15G2R1
13	1	Pasty	*C. ubiquitum*	XIIa
14	1	Diarrhoeic	*C. parvum*	IIaA15G2R1
15	1	Pasty	*C. ubiquitum*	XIIa

## Discussion

The present study describes the diffusion and the intensity of *Cryptosporidium* infection in apparently healthy and in diarrhoeic adult sheep and lambs in Italy. In addition, it updates the knowledge on the epidemiology and molecular identification of *Cryptosporidium* spp. in sheep farms in Italy since the only previous survey was performed more than 10 years ago and only in lambs (Paoletti et al., 2009).

The overall prevalence of *Cryptosporidium* infection found in this study (10.1%) is higher than that reported in Spain (5.9%; Díaz et al., 2018a) and lower than most investigations worldwide, which have reported infection rates ranging from 14 to 29% (Holsback et al., 2018, Mi et al., 2018, Chikweto et al., 2019, Khan et al., 2019). A prevalence higher than 50% has also been recorded (Bhat et al., 2019). These variations in the prevalence may be due to sample size, geographical region, climate, age of animals, diagnostic methods used, breed, hygiene conditions and management practices (Santin, 2020). In addition, given the low and intermittent oocyst shedding of healthy animals, the sensitivity of microscopical techniques for detecting *Cryptosporidium* oocysts (ranging from 37.0% to 90.8%) and given that a single sample per animal was analysed in the present study, the percentage of *Cryptosporidium*-positive lambs and ewes may have been underestimated (Ghoshal et al., 2018, Ahmed and Karanis, 2018).

The prevalence found in lambs (16.4%) from Sardinia was similar to that recently reported in lambs in Turkey by both microscopic and molecular methods (19.4%; Kabir et al., 2020) and to that reported by enzyme-linked immunosorbent assay (ELISA) in the only survey carried out on lambs in central Italy (17.45%; Paoletti et al., 2009). However, these studies used different diagnostic methods used in terms of sensitivity and specificity (Ahmed and Karanis, 2018, Santin, 2020). Nevertheless, the prevalence that we found in lambs is in line with studies performed in Belgium (Geurden et al., 2008) and Algeria (Baroudi et al., 2018), with percentages close to 15%. In contrast, other investigations reported higher prevalence rates in lambs, ranging from 19.4 to 29.5% (Papanikolopoulou et al., 2018, Khan et al.2019, Kabir et al., 2020, Rabee et al., 2020).

From an epidemiological point of view, it is worth highlighting that in most farms we found *Cryptosporidium* oocysts exclusively in the lamb group (61.9% of positive farms). Our data revealed that lambs, especially those excreting diarrhoeic faeces, are the most important reservoirs for the protozoan, in line with several other studies (Papanikolopolou et al., 2018, Mammeri et al., 2019, Rabee et al., 2020).

Our data showed that *Cryptosporidium* prevalence rates decreased with faecal consistency and that the highest percentage and oocyst counts were in diarrhoeic faeces. However, these differences were only statistically significant in pre-weaned animals where the risk of being positive to *Cryptosporidium* spp. increased twofold for lambs excreting pasty faeces and more than sixfold for those with diarrhoeic faeces.

These results are in line with many studies that show that cryptosporidial infections are related to neonatal diarrhoea outbreaks in sheep (Muñoz et al., 1996, Quílez et al., 2008, Papanikolopoulou et al., 2018). In contrast, we found that the elimination of diarrhoeic faeces was sporadic in ewes, since only 0.3–0.7% of adult sheep showed diarrhoea. These results confirm findings that cryptosporidial infections are related to the appearance of diarrhoea outbreaks in neonatal lambs and not in adult sheep (Muñoz et al., 1996, Quílez et al., 2008, Papanikolopoulou et al., 2018; Santin, 2020).

Our results also revealed that the prevalence and MI were higher in lambs than in ewes; in fact young animals seem to be more susceptible to infection than adults (Castro-Hermida et al., 2011, Cacciò et al., 2013, Mi et al., 2018, Santin et al., 2020).

Although the prevalence and MI in sheep before and after parturition were lower than in lambs, adults produce a large volume of faeces and thus may be responsible for environmental contamination with *Cryptosporidium* oocysts (Ortega-Mora et al., 1999, Paraudand Chartier, 2012, Chikweto et al., 2019). In addition, adult sheep presenting low parasite burdens may spread an amount of oocysts below the threshold value of detection shown by microscopy (Ghoshal et al., 2018). Our results thus suggest that ewes in the peripartum period, and especially sheep after parturition since they showed higher MI values, may represent a reservoir of potential pathogenic *Cryptosporidium* species for lambs (Ortega-Mora et al., 1999; Castro-Hermida et al., 2005, 2007; Firoozi et al., 2019). In fact, lambs are weaned 30–40 days after birth in Sardinia, and, therefore, a high risk of maternal transmission of the protozoan to lambs may occur. Sharing the same night shelter and the natural suckling reported in the present survey may contribute to the transmission of *Cryptosporidium* infection among all animal groups. However, previous studies on domestic ruminants suggest that adults do not play a key role in the transmission of pathogenic species to newborn animals (Fayer et al., 2007; Díaz et al., 2018b). Unfortunately our attempt at molecular identification of the *Cryptosporidium* species in adult sheep was unsuccessful.

Molecular analysis of faecal specimens positive to microscopy was, however, successful in a few samples, above all in diarrhoeic lambs. Although PCR has been found to be the most sensitive technique for detecting *Cryptosporidium* infections (Santín et al., 2020), some studies have reported lower infection rates using molecular methods, especially in samples containing a low number of oocysts (Fayer et al., 2007, Mueller-Doblies et al., 2008, Díaz et al., 2010). This is in line with our findings since amplification was only achieved in diarrhoeic samples containing high numbers of oocysts.

The two *Cryptosporidium* species identified in lambs from Sardinia, *C. parvum* and *C. ubiquitum*, are considered the most common species in sheep together with *C. xiaoi* (Ye et al., 2013, Tzanidakis et al., 2014, Li et al., 2016, Baroudi et al., 2018, Díaz et al., 2018a, Mi et al., 2018, Mammeri et al., 2019, Qi et al., 2019, Rabee et al., 2020, Santín, 2020). Our results are in agreement with most investigations reporting *C. parvum* as the main species in pre-weaned lambs (Castro-Hermida et al., 2007, Paoletti et al., 2009,Tzanidakis et al., 2014) and especially in those presenting diarrhoea (Mueller-Doblies et al., 2008, Quílez et al., 2008, Díaz et al., 2010, 2015, Papanikolopoulou et al., 2018, Mammeri et al., 2019, Kabir et al., 2020). However, *C. parvum* has also been commonly found in healthy lambs and even adults (Díaz et al., 2018a, Majeed et al., 2018). Findings from other countries in Europe indicate that *C. parvum* is also the predominant species in post-weaned and adult sheep (Castro-Hermida et al., 2007, Mueller-Doblies et al., 2008, Díaz et al., 2018a).

*Cryptosporidium ubiquitum* and *C. xiaoi* are often the most common species in older animals (Santin, 2020) and are considered as low-pathogenic species (Fayer and Santín, 2009, Fayer et al., 2010) and therefore are mostly reported in apparently healthy sheep (Díaz et al., 2018a, Majeed et al., 2018, Qi et al., 2019). In fact, our results showed the presence of *C. ubiquitum* in apparently healthy lambs and only in one diarrhoeic lamb in which the diarrhoea may have been caused by an infectious agent not investigated in this study. Since no isolates from ewes were genotyped, this may be the reason why we only found few cases of *C. ubiquitum* and none of *C. xiaoi*.

The subtyping analysis identified *C. parvum* subtypes belonging to the allelic family IIa, which has already been reported in calves and sheep and is sometimes responsible for human infections (Quílez et al., 2008, Díaz et al., 2010, 2018b, Kabir et al., 2020), as well as IId, previously reported in Europe (Quílez et al., 2008, Papanikolopoulou et al., 2018). In fact, small ruminants are infected exclusively with *C. parvum* subtypes that belong to the IIa and IId allelic families (Squire et al., 2017). Both *C. parvum* subtypes IIa and IId have also been found in cattle in Sardinia (Díaz et al., 2018b).

Although in our study there was no close proximity to cattle for any of the sheep farms examined, there may still have been some cross transmission, as has been reported in Sardinia for other parasites (Scala et al., 2017). *Cryptosporidium ubiquitum* subtyping studies are currently very limited; however, the subtype XIIa identified in lambs from Sardinia has been already recognized as the predominant subtype in small ruminants worldwide (Li et al., 2014).

Our findings have public health implications since both the *Cryptosporidium* species identified are considered zoonotic (Xiao, 2010). *C. parvum* is considered the second-most common *Cryptosporidium* species infecting humans (Xiao, 2010). In addition, the *C. parvum* subtype IIaA15G2R1 has been reported to be the most common in humans in Europe (Cacciò and Chalmers, 2016; Feng et al., 2018; Chalmers et al., 2019) and was reported in an AIDS patient in Italy (Del Chierico et al., 2011).

*C. ubiquitum* is a recognized emerging human pathogen, and the subtype that we found (XIIa) has also been related to human infections (Li et al., 2014). Direct contact with sheep has been reported as one of the most frequent causes of zoonotic transmission, together with the consumption of water contaminated with *Cryptosporidium* oocysts excreted by sheep (Robertson, 2009, Li et al., 2014; WHO/FAO, 2014). A study carried out in Italy identified diarrhoeic lambs as the source of zoonotic *C. parvum* for an infant aged 18 months (Cacciò et al., 2013). The risk of zoonotic transmission from sheep is thus higher for those in close contact with sheep, such as farmers and veterinarians.

Our study also highlights the need to establish adequate control and monitoring programs for *Cryptosporidium* infection in sheep farms, primarily through coprological monitoring in lambs. Currently, the only officially registered drug against cryptosporidiosis in sheep is paromomycin sulphate (Hameed et al., 2019); however, it was only authorized in Italy for trade in 2019 (Gazzetta Ufficiale 2019). The control of environmental contamination should be monitored in association with the control of *Cryptosporidium* infections on farms, considering the high resistance of *Cryptosporidium* oocysts in the environment and at normal water treatments (Santin, 2020).

In conclusion, our study revealed that *Cryptosporidium* is a prevalent and widespread parasite in sheep in Sardinia, which hosts almost 45% of the entire Italian sheep stock. Pre-weaned lambs, especially those showing diarrhoea, are shedders of zoonotic and pathogenic *Cryptosporidium* species, and thus, improvements in management and hygiene practices are needed to prevent the transmission of the protozoan. A notable percentage of adult sheep also excreted *Cryptosporidium* oocysts; however, further research is needed to clarify their role in the epidemiology of human and animal cryptosporidiosis.
